# Revealing the roles of GORK channels and NADPH oxidase in acclimation to hypoxia in Arabidopsis

**DOI:** 10.1093/jxb/erw378

**Published:** 2016-10-19

**Authors:** Feifei Wang, Zhong-Hua Chen, Xiaohui Liu, Timothy D Colmer, Lana Shabala, Anya Salih, Meixue Zhou, Sergey Shabala

**Affiliations:** 1 School of Land and Food, University of Tasmania, Hobart, Tasmania, Australia; 2 School of Science and Health, Hawkesbury Institute for the Environment, Western Sydney University, Penrith, NSW, Australia; 3 School of Light Industry Engineering, Guizhou Institute of Technology, Guiyang, China; 4 School of Plant Biology and Institute of Agriculture, The University of Western Australia, Crawley, WA, Australia

**Keywords:** Calcium, epidermis, fluorescence dyes, H_2_O_2_, homeostasis, potassium, reactive oxygen species, signalling, stele, superoxide

## Abstract

Regulation of root cell K^+^ is essential for acclimation to low oxygen stress. The potential roles of GORK (depolarization-activated guard cell outward-rectifying potassium) channels and RBOHD (respiratory burst oxidase homologue D) in plant adaptive responses to hypoxia were investigated in the context of tissue specificity (epidermis versus stele; elongation versus mature zone) in roots of Arabidopsis. The expression of *GORK* and *RBOHD* was down-regulated by 2- to 3-fold within 1 h and 24 h of hypoxia treatment in Arabidopsis wild-type (WT) roots. Interestingly, a loss of the functional GORK channel resulted in a waterlogging-tolerant phenotype, while *rbohD* knockout was sensitive to waterlogging. To understand their functions under hypoxia stress, we studied K^+^, Ca^2+^, and reactive oxygen species (ROS) distribution in various root cell types. *gork1-1* plants had better K^+^ retention ability in both the elongation and mature zone compared with the WT and *rbohD* under hypoxia. Hypoxia induced a Ca^2+^ increase in each cell type after 72 h, and the increase was much less pronounced in *rbohD* than in the WT. In most tissues except the elongation zone in *rbohD*, the H_2_O_2_ concentration had decreased after 1 h of hypoxia, but then increased significantly after 24 h of hypoxia in each zone and tissue, further suggesting that RBOHD may shape hypoxia-specific Ca^2+^ signatures via the modulation of apoplastic H_2_O_2_ production. Taken together, our data suggest that plants lacking functional GORK channels are more capable of retaining K^+^ for their better performance under hypoxia, and that RBOHD is crucial in hypoxia-induced Ca^2+^ signalling for stress sensing and acclimation mechanism.

## Teaser

Knocking out GORK channels prevents K^+^ leak and increases waterlogging tolerance, while reduced RBOHD activity interferes with Ca^2+^ signalling, resulting in a hypoxia-sensitive phenotype.

## Introduction

The increasing frequency of flooding in southeast Asia, east Africa, and southern India (Hirabayashi *et al.*, 2013) has led to large areas of farmlands worldwide adversely influenced by waterlogging stress on a regular basis (Shabala, 2011; Voesenek and Bailey-Serres, 2015). Waterlogging occurs as a consequence of high rainfall, superfluous irrigation supplies, and poor soil drainage, imposing major constraints on root respiration and nutrient absorption, with a major impact on crop yield (Bailey-Serres and Colmer, 2014; Herzog *et al.*, 2016; Shabala *et al.*, 2016).

Potassium homeostasis is essential to mediate plant adaptive responses to a broad range of abiotic stresses including hypoxia (Barrett-Lennard and Shabala, 2013; Anschutz *et al.*, 2014). Cytosolic K^+^ participates in many defence-related processes under hypoxic stress, such as detoxifying reactive oxygen species (ROS), preventing cytosolic acidification, and possibly formation of aerenchyma (Shabala *et al.*, 2014). Various abiotic and biotic stresses induce massive K^+^ efflux from root cells; in most cases, this efflux is mediated by depolarization-activated outward-rectifying K^+^ (GORK) channels (Becker *et al.*, 2003; Tran *et al.*, 2013). The GORK channel is expressed in guard cells (hence, the name), root outer cell layers (epidermal cells, root hairs, and cortex), and cells of the phloem tissue (Tran *et al.*, 2013). Both activity and expression levels of GORK channels are significantly affected by stress conditions. Drought-induced elevation in the abscisic acid (ABA) level up-regulates both the channel activity and transcript level of GORK for stomatal closure and drought tolerance (Jeannette *et al.*, 1999; Becker *et al.*, 2003; Chen *et al.*, 2015*b*). The GORK channel comprises a major pathway for salt-induced K^+^ leak in many plant systems (Shabala *et al.*, 2006*b*; Shabala and Cuin, 2008; Jayakannan *et al.*, 2013), and the Arabidopsis *gork1-1* mutant displayed lower activity of proteases and endonucleases for cell death under salinity stress than the wild type (WT; Demidchik *et al.*, 2010). A positive correlation between root K^+^-retaining ability and hypoxia tolerance was also reported for barley (Pang *et al.*, 2006; Zeng *et al.*, 2014). However, the exact role of GORK channels in K^+^ homeostasis under hypoxic stress is still elusive.

Hypoxic stress interferes with the mitochondrial respiration pathway where it leads to the saturation of redox chains, accumulation of NAD(P)H, decreased synthesis of ATP (He *et al.*, 2015; Najeeb *et al.*, 2015; Voesenek and Bailey-Serres, 2015), and generation of ROS (Bailey-Serres and Mittler, 2006; Yamauchi *et al.*, 2014). While ROS are important signalling molecules mediating a broad range of developmental and adaptive responses (Apel and Hirt, 2004; Mittler *et al.*, 2004; De Pinto *et al.*, 2012; Rodrigo-Moreno *et al.*, 2013), accumulation of high amounts of ROS can be detrimental to cells. Hydrogen peroxide (H_2_O_2_) and superoxide (O_2_·^−^) are both produced by a number of intracellular and extracellular sources (Blokhina *et al.*, 2001; Suzuki *et al.*, 2012; Wudick and Feijo, 2014). One of these sources are plant respiratory burst oxidase homologues (RBOHs) that oxidize NADPH and transfer the electron to oxygen to generate superoxide anions in the apoplastic space (Sagi and Fluhr, 2006). RBOHs contain six conserved transmembrane domains at the plasma membrane and cytosolic NADPH- and FAD-binding domains in the C-terminal region. They also contain two calcium-binding EF-hands and phosphorylation domains in the N-terminal regulatory region (Torres and Dangl, 2005; Suzuki *et al.*, 2011). A survey of gene expression of *RBOH* genes in Arabidopsis identified that *RBOHD* is significantly up-regulated after 6 h and 12 h of anoxia (Yang and Hong, 2015). Also, a *rbohD* knockout mutant showed reduced superoxide production and low H_2_O_2_ accumulation during defence responses (Torres *et al.*, 2002) and poor survival under anoxia (Pucciariello *et al.*, 2012; Chen *et al.*, 2015*a*), suggesting a role for RBOHD under anoxic stress. The mechanisms for this increased sensitivity to anoxia remain unclear. It is known that ABA levels increase significantly in plants when subjected to soil waterlogging (Zhang and Davies, 1987; Castonguay *et al.*, 1993). In Arabidopsis guard cells, hyperpolarization-activated Ca^2+^-permeable channels were activated by H_2_O_2_, and suppression of NAD(P)H oxidases partially inhibited ABA-induced stomatal closure (Pei *et al.*, 2000). H_2_O_2_ also activates a range of cation-permeable non-selective cation channels (Demidchik *et al.*, 2003, 2007; Mori and Schroeder, 2004; Ordonez *et al.*, 2014), thus affecting intracellular K^+^ and Ca^2+^ homeostasis and signalling (Shabala and Pottosin, 2014). Also, by interacting with transition metals, either in the apoplast (Demidchik, 2015; Demidchik *et al.*, 2007) or in the cytosol (Rodrigo-Moreno *et al.*, 2013), H_2_O_2_ can form hydroxyl radicals, that in turn directly activate GORK channels (Demidchik *et al.*, 2014).

The roles and interaction between GORK channels and NADPH oxidase in hypoxic responses of roots requires elucidation since both can impact cellular signalling and K^+^ homeostasis and therefore possibly acclimation to hypoxia. Moreover, the tissue-specific context of these hypoxia responses in roots needs to be investigated. In this work, we have studied profiles of K^+^, Ca^2+^, superoxide, and H_2_O_2_ distribution in specific tissues (epidermis and stele) and zones (elongation and mature) in Arabidopsis roots in the WT, and *gork1-1* and *rbohD* mutants. We report that the loss of a functional GORK channel in Arabidopsis leads to better K^+^ retention and results in a more hypoxia-tolerant phenotype. We also show that NADPH oxidase activity affects Ca^2+^ distribution and functions in Ca^2+^ signalling under hypoxia stress.

## Materials and methods

### Plant materials and treatments

*Arabidopsis thaliana* WT Columbia-0 (Col-0) and two loss-of-function mutants *gork1-1* (SALK_082258) and *rbohD* (SALK_021661) (both in Col-0 background) were obtained from the Arabidopsis Biological Resource Centre (http://www.Arabidopsis.org/abrc/). Seeds were surface-sterilized by 20% commercial bleach [1% (v/v) NaClO] for 10 min, washed with sterilized distilled water five times, and then stratified in fresh distilled water at 4 °C for 2 d. Seeds were then sown in Petri dishes containing 1% (w/v) phytogel, half-strength Murashige and Skoog (MS) medium, and 0.5% (w/v) sucrose at pH 5.7, sealed with 3M micro-pore tape (3M Health Care, St. Paul, MN, USA), and then placed in a growth chamber and positioned vertically to allow root growth along the surface of the medium. The conditions in the growth chamber were 16 h/8 h light/dark cycles, with 100 µmol m^−2^ s^−1^ photon flux density during the light period, and temperature at 22 °C. Unless specified, all chemicals were of analytical grade from Sigma-Aldrich (Castle Hill, NSW, Australia).

Hypoxic treatment was imposed by submerging the 10-day-old Arabidopsis seedlings in 0.2% (w/v) agar solution pre-bubbled with high purity N_2_ (Coregas, Sydney, NSW, Australia) (Pang *et al.*, 2006; Wang *et al.*, 2016). For gene expression analysis, WT seedlings were treated with hypoxia for 1, 24, and 72 h. The treatment solution contained 10 mM KCl, 5 mM Ca^2+^-MES, pH 6.1, and 0.2% (w/v) agar which was dissolved by boiling while being stirred, and the solution was cooled down to room temperature, and then bubbled with high purity N_2_.The whole seedlings were submerged in agar solution in a transparent container and were exposed to 16 h/8 h light/dark cycles, enabling plant photosynthetic activity; this approach was taken following earlier work on Arabidopsis (Mustroph *et al.*, 2009). The addition of agar prevents convective movements in the solution and so impedes re-entry of any O_2_ during the treatment period (Colmer *et al.*, 2004). For confocal imaging of K^+^, Ca^2+^, superoxide, and H_2_O_2_ distribution in roots, the WT and two mutants were subjected to hypoxic treatment for 1, 24, and 72 h. The whole seedlings were submerged in the pre-bubbled agar solution under a photon flux density of 100 µmol m^−2^ s^−1^ and at 22 °C. To better analyse the K^+^ and Ca^2+^ changes in plant, the whole seedlings were exposed to K^+^-free or Ca^2+^-free agar solution in hypoxia. When measuring the K^+^ concentration within tissues using a dye (see below), the hypoxic solution contained 5 mM NaCl, 5 mM Ca^2+^-MES, pH 6.1, with 0.2% (w/v) agar. For Ca^2+^ measurement, again using a dye (see below), the hypoxic solution was 10 mM KCl, 5 mM Na^+^-MES, pH 6.1, with 0.2% (w/v) agar. For ROS measurement, the hypoxic solution was 10 mM KCl, 5 mM Ca^2+^-MES, pH 6.1, with 0.2% (w/v) agar. Seedlings used for control were exposed to air under the same light and temperature regime.

### Whole-plant physiological assessment

In the phenotyping experiments, stratified *gork1-1*, *rbohD*, and WT seeds were sown in 0.2 litre pots filled with peat moss, perlite, vermiculite, and coarse sand (2:1:1:1, v/v), and grown for 3 weeks at 21 °C using a 12/12 h light [photosynthetically active radiation (PAR) 100 µmol m^−2^ s^−1^]/dark regime. Waterlogging treatment was implemented by immersing pots of 3-week-old plants at water level 0.5 cm above the soil surface and leaving them for another 3 weeks. The above-ground biomass was determined as fresh weight per plant. Leaf chlorophyll content was measured with a SPAD meter (SPAD-502, Minolta, Japan). Measurements were taken for at least six biological replicates for each treatment in a single experiment.

### Quantitative real-time PCR

Total RNA was extracted from roots of 10-day-old Arabidopsis WT (Col-0) with TRIZOL reagent (Life Technologies, Mulgrave, VIC, Australia) according to Chen *et al.* (2015*b*) and Liu *et al.* (2014). RNA was reverse transcribed with a sensiFAST Kit (Bioline, Alexandria, NSW, Australia) and real-time PCR was performed with a SensiMix SYBR No-ROX Kit (Bioline, Alexandria, NSW, Australia) using a Rotor-Gene Q6000 (QIAGEN, Hilden, Germany). The gene-specific primers are as below: forward 5'-CACTATGGCAACTGTCGGTTATGG-3' and reverse 5'-GCGGTTCATGAAACTTATGAGATC-3' for *GORK*; and forward 5'-TGCGGGTGCCCATTTAAC-3' and reverse 5'-CTTTCACAAACCACCAGTAGC-3' for *RBOHD*. Amplification of the RNA polymerase II subunit (*RPB2*) (forward primer 5'-TTCCCCGTTCCGATAACT-3' and reverse primer 5'-ATGCTCTGCCGTCCACC-3') gene was used as an internal control. The PCR program was two steps: one cycle of 95 °C, 10 min; 40 cycles of 95 °C, 15 s; 60 °C, 15 s; 72 °C, 15 s. The amplification of the target genes was monitored every cycle by SYBR-green fluorescence. Three biological and three technical replicates were performed for each treatment.

### Ion concentration measurements

The Calcium Green-5N, AM (Invitrogen, Eugene, OR, USA) and Asante Potassium Green-2 (APG-2, TEFLabs, Austin, TX, USA) dyes were employed to measure the Ca^2+^ and K^+^ concentration, respectively, in Arabidopsis root cells. The optimal concentrations of Calcium Green-5N and APG-2 dye were established in our previous work (Wang *et al.*, 2016). Then, 20 µM APG-2 or 15 µM Calcium Green-5N AM were added to K^+^ (5 mM NaCl, 5 mM Ca^2+^-MES, pH 6.1) or Ca^2+^ (10 mM KCl, 5 mM Na^+^-MES, pH 6.1) measuring buffers, respectively. After 1, 24, and 72 h of hypoxic treatment, Arabidopsis seedlings were incubated in the dye-containing measuring buffers for 3 h in the dark at room temperature. They were then washed in distilled water for 3 min to remove the residual dyes before measuring the fluorescence intensity. For each treatment, at least nine biological replicates (individual roots) were measured; for each of them, between 20 and 30 cells (technical replicates) were averaged.

A confocal laser scanning microscope (SP5, Leica Microsystems, Heidelberg, Germany) was used to measure fluorescent signals from roots, as described in detail in Bonales-Alatorre *et al.* (2013). APG-2 and Calcium Green-5N fluorescence emissions were detected in the photomultiplier at 530–550 nm and 520–550 nm, respectively. Fluorescent images were analysed with Image J software (NIH, USA) based on integrated density with subtraction of the background signal which was measured from an empty region of similar size (Wang *et al.*, 2016). The cytosolic and vacuolar K^+^ concentration in Arabidopsis root cells was also determined using the fluorescent dye APG-2. For imaging analysis, several lines were drawn across the tissue region of interest in the elongation and mature zones with Leica Application Suite X software (Leica Microsystems, Heidelberg, Germany). For each treatment, at least nine biological replicates (individual roots) were measured; for each of them ~60 cells (technical replicates) were averaged. Continuous fluorescence was quantified in arbitrary units by LAS AF software based on intensity (Wu *et al.*, 2015). For reporting purposes, the relative total cell K^+^ and Ca^2+^ concentration data shown in Figs 4 and 5 was divided by 1000.

### K^+^ flux measurement

Net K^+^ flux was measured using the MIFE (non-invasive microelectrode ion flux estimation; University of Tasmania, Hobart, Australia) technique. The specific details relating to the MIFE theory and electrode fabrication and calibration are available in our previous publications (Newman, 2001; Shabala *et al.*, 2006*a*). Arabidopsis seedlings were grown in a Petri dish for 10 d and then the whole Petri dish was submerged vertically in BSM solution (0.5 mM KCl+0.1 mM CaCl_2_, pH 5.6) with 0.2% agar pre-bubbled with N_2_ in a sealed plastic container for 3 d under a photon flux density of 100 µmol m^−2^ s^−1^ and at 22 °C. Prior to the MIFE measurement, one Arabidopsis root was cut into 2 cm long segment from the tip and then immobilized horizontally in a 10 ml Perspex measuring chamber (10.5 × 0.8 × 2.0 cm). For treatment, 9 ml of BSM solution with 0.2% agar pre-bubbled with N_2_ were added to the chamber at 21 °C. For the control, roots were exposed only to BSM solution. Net K^+^ flux was measured by microelectrodes in the elongation zone (0.4 mm from the root tip) and mature zone (5 mm from the root tip) 2 h after the treatment. The net ion flux between the two positions was recorded by MIFE CHART software and calculated by the MIFEFLUX program. For each treatment and two different zones, at least 12 replicates were measured for each treatment.

### ROS measurements

The H_2_O_2_-sensitive fluorescent probe cell-permeant 2',7'-dichlorodihydrofluorescein diacetate (H_2_DCFDA or DCF, Molecular Probes, Eugene, OR, USA) and superoxide fluorescent dye dihydroethidium (DHE) AM ester (Invitrogen, Eugene, OR, USA) were employed to measure H_2_O_2_ and superoxide concentration in Arabidopsis root cells, respectively (Chen *et al.*, 2015*b*). The indicators were dissolved in DMSO (Sigma) to a stock concentration of 1 mM. Then, 20 µM DCF or 10 µM DHE were each added to a measuring buffer (10 mM KCl, 5 mM Ca^2+^-MES, pH 6.1). After 1, 24, and 72 h of hypoxic treatment, Arabidopsis seedlings were incubated in the dye-containing measuring buffers for 30 min in the dark. The stained seedlings were washed in distilled water for 3 min to remove residual dyes before measuring fluorescence intensity in elongation epidermal, elongation stelar, mature epidermal, and mature stelar root cells. The fluorescence images of superoxide and H_2_O_2_ were collected with excitation at 488 nm and 514 nm for DCF and DHE, respectively, and emission at 517–527 nm for DCF and 590–630 nm for DHE. Then, fluorescent images were analysed with Image J software (NIH, USA) based on integrated density similar to ion concentration measurements. For each treatment and ROS species, at least nine biological replicates (individual roots) were measured; for each of them, between 20 and 30 cells (technical replicates) were averaged. Relative total cell superoxide and H_2_O_2_ concentration data shown in Figs 9 and 10 were divided by 1000.

### Statistical analysis

Statistical analysis was performed using IBM SPSS Statistics 21 (IBM, New York, NY, USA). All data in the figures are means ±SE. The significant differences were compared using Duncan’s multiple range test. Different lower case letters represent significant difference between genotypes and/or treatments at *P<*0.05. The statistical significance of phenotypes in the control and waterlogging treatment was tested by paired samples *t*-test. The significance levels are *P<0.05, **P<0.01, and ***P<0.001.

## Results

### *Hypoxia affects* GORK and RBOHD *transcript levels*

**Fig. 1.**
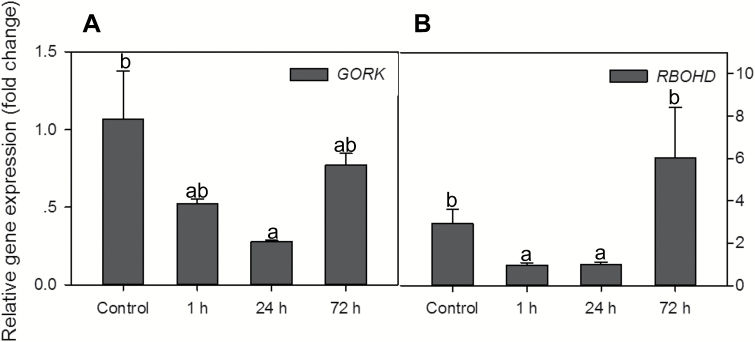
Relative expression of *GORK* and *RBOHD* in roots of Arabidopsis wild-type (Col-0) after 1, 24, and 72 h of hypoxia. *RPB2* (RNA polymerase II subunit) was used as a reference gene. Data are the mean ±SE (*n*=3 separate experiments each involving 3–5 biological replicates). Different lower case letters indicate a significant difference between treatments at *P<*0.05.

We first investigated the effects of hypoxia on expression of *GORK* and *RBOHD* in roots of 10-day-old WT Arabidopsis after 1, 24, and 72 h of hypoxic treatments (Fig. 1). Compared with the control, *GORK* and *RBOHD* were both down-regulated within 1 h and 24 h of hypoxia treatments, and then up-regulated after 72 h of hypoxia treatment (Fig. 1A, B). *GORK* transcript levels were reduced by 74% compared with the control when exposed to 24 h of hypoxia, and the expression of *RBOHD* was increased by 2-fold after 72 h of hypoxia (Fig. 1).

### The lack of functional GORK channels results in a waterlogging-tolerant phenotype

**Fig. 2.**
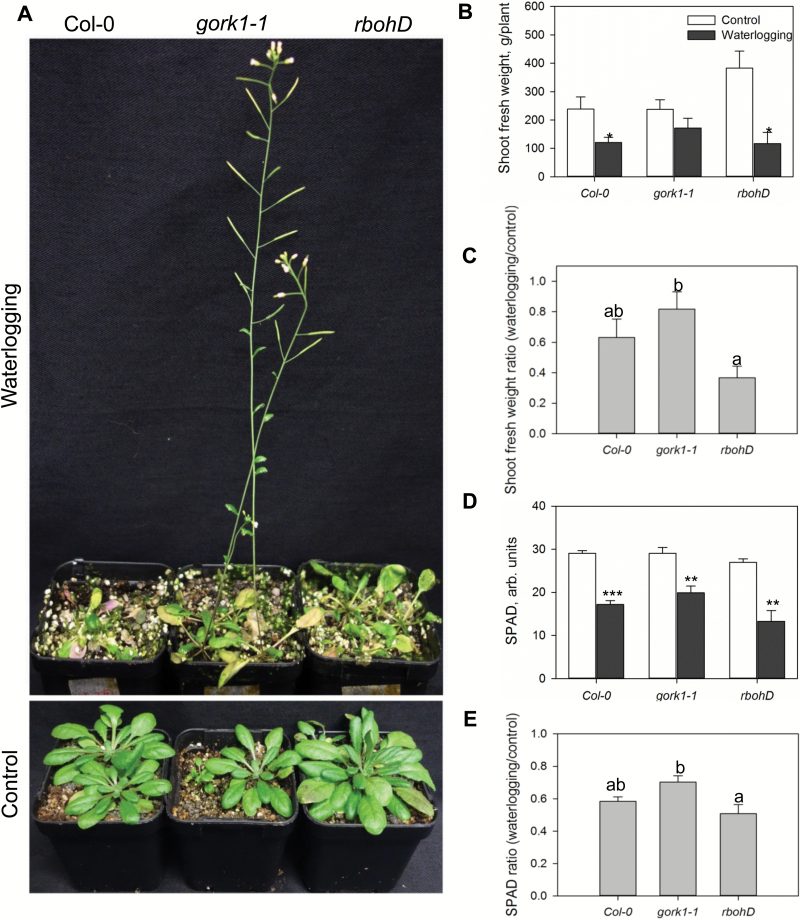
Effects of waterlogging stress on growth and chlorophyll content of 6-week-old Arabidopsis plants. Three-week-old Arabidopsis plants were subjected to waterlogging treatment for a further 3 weeks. Chlorophyll content (SPAD) (D) was measured at the end of the experiment (from 6-week-old plants) and plants were used for morphological (A) and biomass analysis (B). Data are the mean ±SE (*n*=6 biological replicates). The ratios of shoot fresh weight loss (C) and chlorophyll content loss (E) were calculated.

Three weeks of waterlogging stress affected growth of WT, *gork1-1*, and *rbohD* plants although to a different extent (Fig. 2A). Waterlogging stress significantly (*P*<0.05) reduced the shoot fresh weight in the WT and *rbohD*, but *gork1-1* plants showed a waterlogging-tolerant phenotype (Fig. 2B). In *gork1-1,* waterlogging reduced shoot fresh weight by 18% relative to control, while in the WT and *rbohD* this reduction was 37% and 63%, respectively (Fig. 2B, C). The leaf chlorophyll content (SPAD value) was also significantly affected by waterlogging treatment (Fig. 2D), with relative SPAD values being reduced by 49, 42, and 30% in *rbohD*, the WT, and *gork1-1*, respectively (Fig. 2E). The genotypic difference in tolerance to the waterlogging stress was also clearly visible in the fact that *gork1-1* formed robust and well-developed siliques after 3 weeks of waterlogging stress, while the WT and *rbohD* did not show any inflorescences at all (Fig. 2A).

### Hypoxia-induced K^+^ and Ca^2+^ distributions show tissue and zone specificity in Arabidopsis mutants

**Fig. 3.**
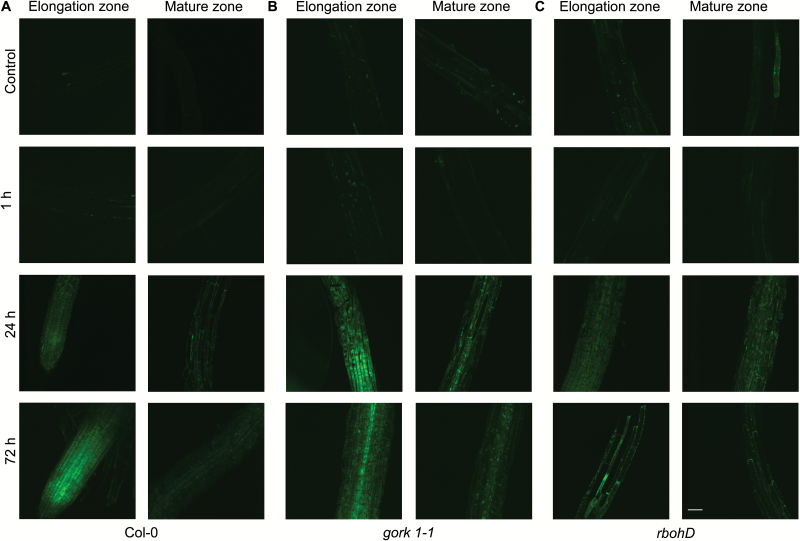
Effect of hypoxic stress on K^+^ distribution in the root elongation and mature zone in Arabidopsis wild type (Col-0), *gork1-1*, and *rbohD*. Representative images of the root elongation and mature zone in Col-0 (A), *gork1-1* (B), and *rbohD* (C) under control and hypoxic treatment are shown. Ten-day-old seedlings was stained with K^+^ indicator (Asante Potassium Green-2) and visualized with a confocal imaging system. One out of nine typical images is shown for each line. Scale bar=50 µm.

**Fig. 4.**
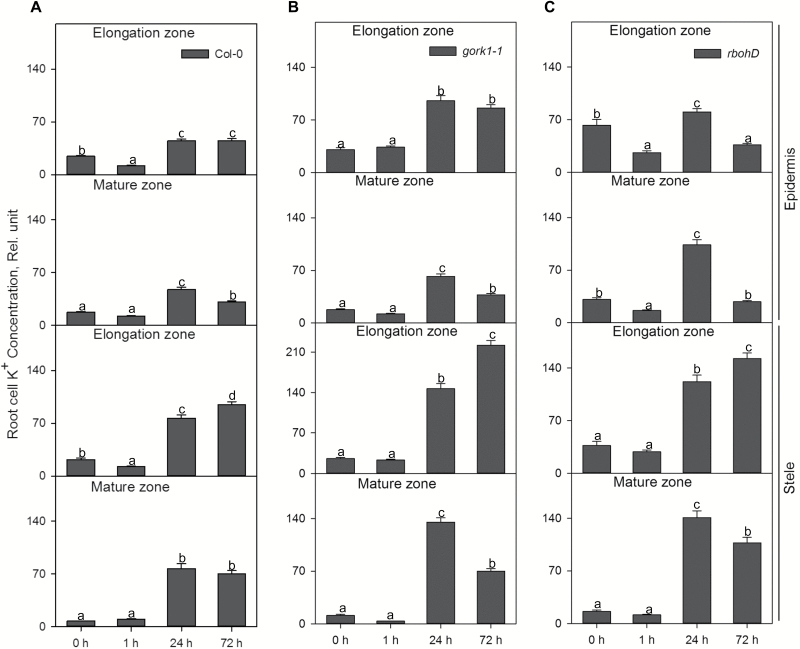
The time dependence of the relative K^+^ concentration in the elongation and mature zones in the root epidermis and stele of Col-0 (A), *gork1-1* (B), and *rbohD* (C) in response to hypoxia. Relative K^+^ concentration was calculated by the fluorescence integrated density using Image J software. Data are the mean ±SE [*n*=180–270; 20–30 cells analysed for at least nine individual roots (biological replicates)]. Different lower case letters indicate a significant difference at *P*<0.05.

In the root epidermis of the WT, hypoxia led to a decline in the K^+^ concentration of the elongation zone, but no changes were observed in the mature zone after 1 h of hypoxia (Figs 3A, 4A). However, as hypoxic treatment progressed, there was a significant increase of K^+^ in the epidermis of both the elongation zone and the mature zone compared with the control. A similar trend was also found in stelar cells in both zones in all genotypes (Fig. 4A–C). The K^+^ concentration in epidermal cells of the elongation zone in *gork1-1* increased significantly (*P<*0.05) after 24 h of hypoxia, but in the mature zone it was decreased after 72 h of hypoxia compared with 24 h (Fig. 4B). Epidermal cells in *rbohD* exhibited a distinct K^+^ distribution. One hour of hypoxia caused a significant decrease of K^+^ accumulation in epidermal cells in both zones in *rbohD* then a significant rise after 24 h, followed by a significant decrease after 72 h of hypoxia (Fig. 4C). Comparing the genotype-specific K^+^ concentration in different root zones and tissues, there was, in general, more K^+^ accumulation in both mutants than in the WT after 24 h of hypoxia (Fig. 4). In *gork1-1*, there were 1.3- to 2.1-fold increases in K^+^ concentration compared with corresponding cells (epidermal cells in the elongation and mature zone; stelar cells in the elongation and mature zone) in the WT; likewise, in *rbohD*, the K^+^ concentration was 1.6- to 2.2-fold higher than that in the WT (Fig. 4). Comparing tissue specificity, in either the elongation zone or the mature zone, all three genotypes had higher K^+^ concentrations in the stele than in the epidermis after 24 h of hypoxia (Fig. 4). In the elongation zone, the K^+^ concentrations in the WT, *gork1-1*, and *rbohD* were 1.7-, 1.5-, and 1.5-fold higher, respectively, in stellar cells than in epidermal cells; in the mature zone, they were 1.6-, 2.2-, and 1.4-fold higher in the stelar cells (Fig. 4).

**Fig. 5.**
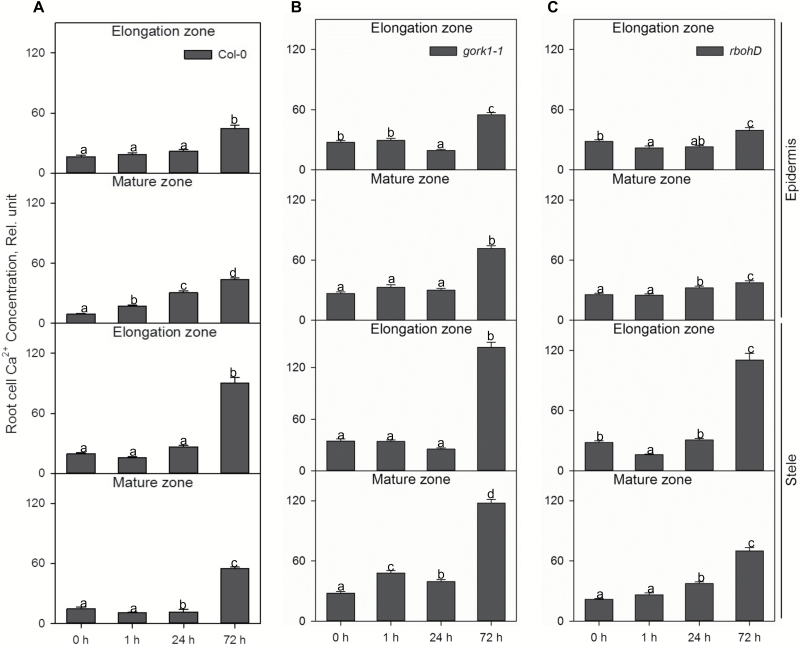
The time dependence of the relative Ca^2+^ concentration in the elongation and mature zone in the root epidermis and stele of Col-0 (A), *gork1-1* (B), and *rbohD* (C) in response to hypoxia. The relative Ca^2+^ concentration was calculated by the fluorescence integrated density using Image J software. Data are the mean ±SE [*n*=180–270; 20–30 cells analysed for at least nine individual roots (biological replicates)]. Different lower case letters indicate a significant difference at *P*<0.05.

Changes in Ca^2+^ concentration in different root zones and tissues showed a time-dependent increase, with 72 h of hypoxia treatment causing the highest increase in Ca^2+^ concentration in the WT, *gork1-1*, and *rbohD* compared with the control after 1 h and 24 h of treatment (Fig. 5, ). In *gork1-1*, epidermal and stelar cells in both zones showed the highest Ca^2+^ concentration after 72 h of hypoxia compared with the WT and *rbohD* (Fig. 5). There was more Ca^2+^ accumulated in stelar cells than in epidermal cells of both zones in all three genotypes (Fig. 5; Supplementary Fig. S1 at *JXB* online). Interestingly, with the clear trend in stress-induced accumulation of Ca^2+^ in each zone and tissue after 72 h of hypoxia, the increase was much less pronounced in *rbohD* than in the WT. In epidermal cells, there were only 1.4- and 1.5-fold increases in *rbohD* between 72 h of hypoxia and control in the elongation and mature zone, respectively. At the same time, in the WT, these increases were 2.7- and 4.8-fold, respectively. In stelar cells, *rbohD* showed 4.0- and 3.3-fold increases and there were 4.6- and 3.7-fold increases in the WT (Fig. 5A, C). Comparing Ca^2+^ concentrations in the epidermis between the WT and mutants, it was shown that in the elongation zone of *rbohD*, the Ca^2+^ content was 12% lower than in the WT, and that of *gork1-1* was 1.2-fold higher than in the WT; in the mature zone of *rbohD*, the Ca^2+^ content was 15% lower than that in the WT and that of *gork1-1* was 1.6-fold higher than that of the WT after 72 h of hypoxia (Fig. 5).

### *Hypoxia induces high K^+^ accumulation in both the cytosol and vacuole of stellar cells in* gork1-1 *and the WT*

**Fig. 6.**
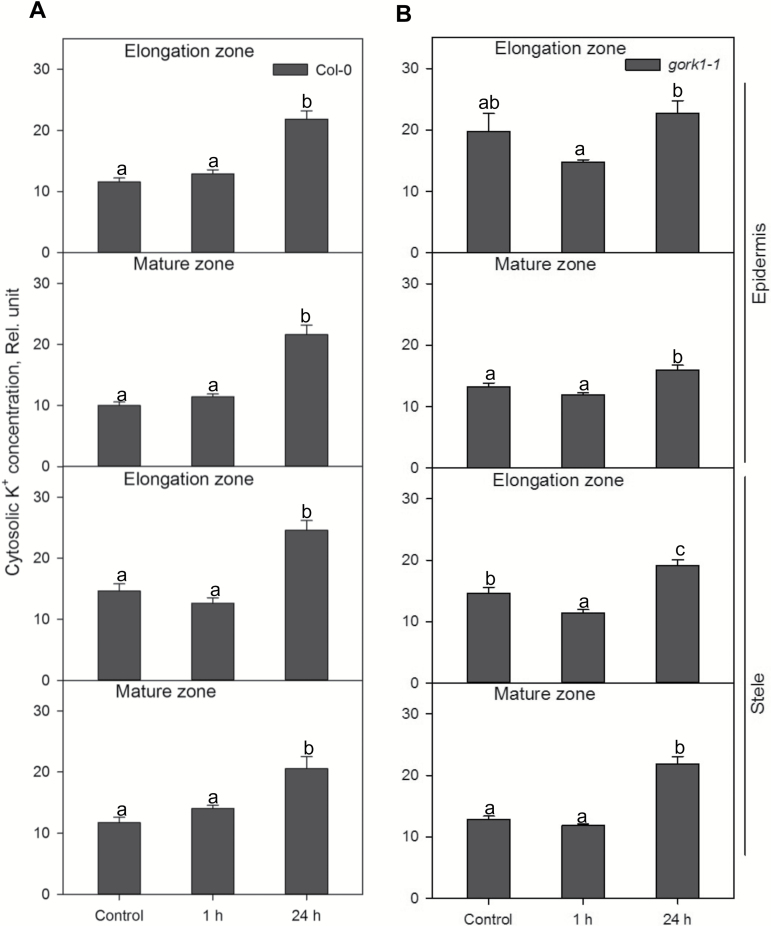
Relative K^+^ concentration in the cytosol in the elongation and mature zone in the root epidermis and stele of Col-0 (A) and *gork1-1* (B) under normoxic or hypoxic treatments for 1 h and 24 h. Data are the mean ±SE [*n*=450–600; 50–60 cells analysed for at least nine individual roots (biological replicates)]. Different lower case letters indicate a significant difference at *P*<0.05.

**Fig. 7.**
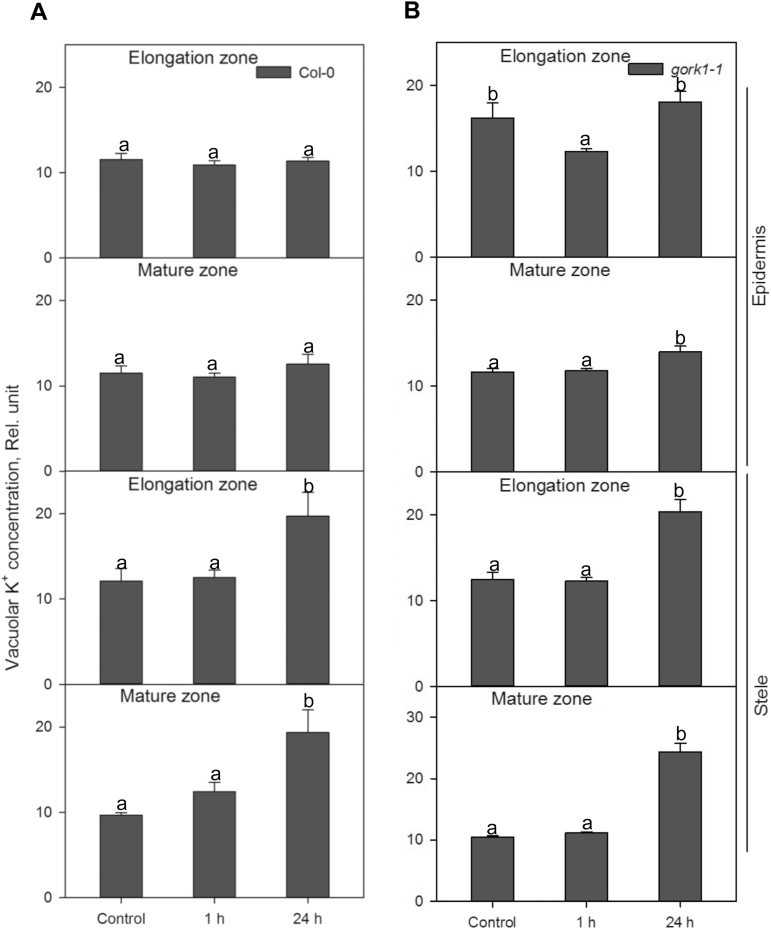
Relative K^+^ concentration in the vacuole in elongation and mature zone in the root epidermis and stele of Col-0 (A) and *gork1-1* (B) under normoxic or hypoxic treatments for 1 h and 24 h. Data are the mean ±SE [*n*=450–600; 50–60 cells analysed for at least nine individual roots (biological replicates)]. Different lower case letters indicate a significant difference at *P*<0.05.

K^+^ concentrations in both the cytosol and vacuole of stelar cells in *gork1-1* and the WT were significantly increased after 24 h of hypoxic stress in both the elongation and mature zones (Figs 6, 7). In the cytosol, up to 2.2-fold higher K^+^ was accumulated in epidermal and stelar cells in both the elongation and mature zone in the WT and *gork1-1* after 24 h of hypoxia (Fig. 6), which was consistent with the trends of the relative total cell K^+^ concentration in the WT and *gork1-1* (Fig. 4A, B; Table 1). After 1 h of hypoxia, all cell types in *gork1-1* and the WT except the stelar cells in the elongation zone of *gork1-1* root showed no significant changes of cytosolic K^+^ between control and 1 h of hypoxia (Fig. 6). The changes in vacuolar K^+^ concentration in the WT and *gork1-1* displayed more tissue specificity (Fig. 7). In the stele of both the elongation zone and mature zone, K^+^ concentrations were increased by 1.5- to 2.0-fold for the WT and by 1.7- to 2.4-fold for *gork1-1* after 24 h of hypoxia (Fig. 7). The epidermis in both the elongation zone and mature zone in the WT revealed no changes after 24 h of hypoxia compared with the control (Fig. 7A); the epidermis in the elongation zone of *gork1-1* also showed no changes, but a significant increase was seen in the mature zone (Fig. 7B).

**Table 1.** Effects of hypoxia on relative K^+^ concentrations in the cytosol and vacuole of the elongation zone epidermal cells and stelar cells, and mature zone epidermal cells and stelar cell in roots of Arabidopsis wild-type (Col-0) and *gork1-1* (ratio of hypoxia ion concentration to control ion concentration) Measurements were collected from elongation epidermal cell, elongation stele cell, mature epidermal cell, and mature stele cell of five Arabidopsis replicates.

	Col-0				*gork1-1*			
	Cytosol		Vacuole		Cytosol		Vacuole	
	1 h	24 h	1 h	24 h	1 h	24 h	1 h	24 h
Elongation epidermis	1.1	2.1	1.0	1.0	0.9	1.2	0.9	1.2
Mature epidermis	1.1	2.2	0.9	1.1	1.0	1.2	1.0	1.1
Elongation stele	0.9	1.6	1.1	1.5	0.8	1.3	1.1	1.7
Mature stele	1.2	1.7	1.3	2.0	1.0	1.8	1.1	2.4

### *The* gork *mutant showed better K^+^ retention than the WT under hypoxic stress*

**Fig. 8.**
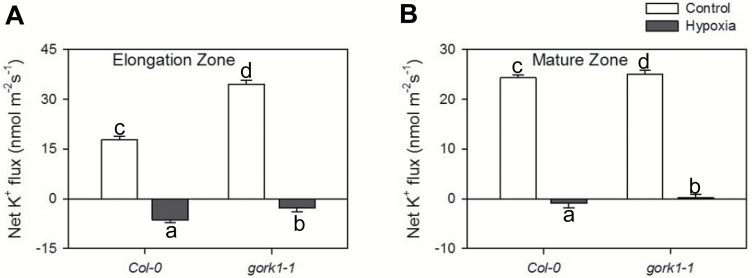
Net K^+^ fluxes measured from the root elongation (A) and the mature (B) zones of Col-0 and *gork1-1* exposed to 72 h of hypoxia. Each bar represents the mean ±SE of 8–12 seedlings. Different lower case letters indicate a significant difference at *P*<0.05.

Steady-state net K^+^ flux was measured from the elongation and mature zones of excised root segments in *gork1-1* and the WT for 5 min (Fig. 8). Under normoxic conditions, both the elongation and mature zone in both the WT and *gork1-1* showed K^+^ influx, while a significant K^+^ loss was induced by 72 h of hypoxia in both root zones and genotypes (Fig. 8A, B). In the elongation zone, hypoxia induced K^+^ efflux from both genotypes, with fluxes of 6.4 nmol m^−2^ s^−1^ from the WT and 2.8 nmol m^−2^ s^−1^ from *gork1-1* from the roots to the medium (Fig. 8A). In the mature zone, the WT showed significant efflux while *gork1-1* maintained a small influx after 72 h of hypoxia (Fig. 8B).

### *Hypoxia induced strong superoxide accumulation in the elongation zone of* rbohD *and more H_2_O_2_ accumulated in the stele than in the epidermis*

**Fig. 9.**
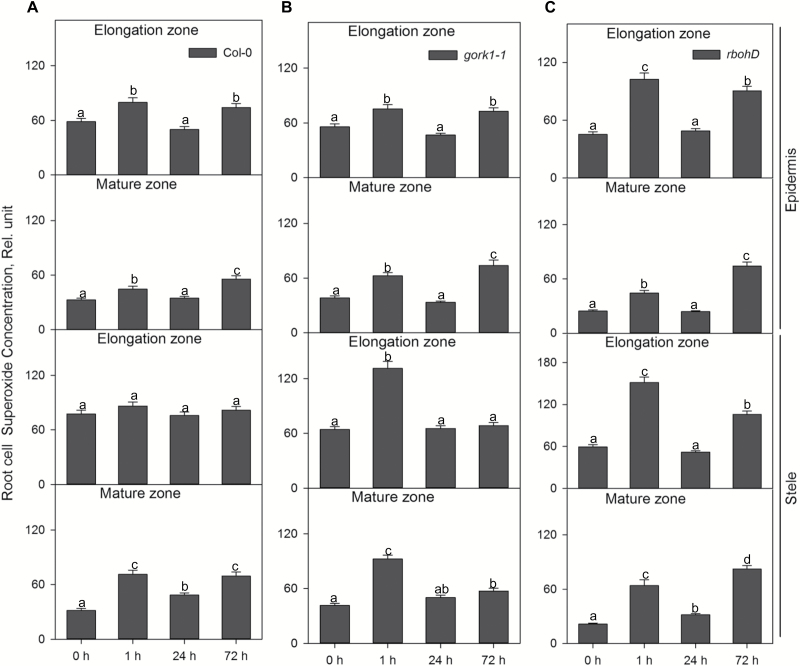
The time dependence of the relative superoxide concentration in the elongation and mature zone in the root epidermis and stele of Col-0 (A), *gork1-1* (B), and *rbohD* (C) in response to hypoxia. Relative superoxide concentration was calculated by the fluorescence integrated density using Image J software. Data are the mean ±SE [*n*=180–270; 20–30 cells analysed for at least nine individual roots (biological replicates)]. Different lower case letters indicate a significant difference at *P*<0.05.

ROS production was measured in different tissues and zones of the root. One hour of hypoxia caused a significant increase in superoxide level in epidermal and stelar cells of both the elongation zone and mature zone in the WT, *gork1-1*, and *rbohD*, except the stelar cells in the elongation zone in the WT (Fig. 9; Supplementary Fig. S2). There was more superoxide accumulated in the elongation zone in the three genotypes than in the mature zone after 1 h and 24 h of hypoxia (Fig. 9). For instance, the superoxide concentration in the epidermis of the elongation zone increased 1.8-fold compared with that of the mature zone in the WT after 1 h of hypoxia (Fig. 9A). In *rbohD*, the superoxide concentration in the stele of the elongation zone was 2.4-fold higher relative to that of the mature zone (Fig. 9C). Interestingly, there was remarkable increase of superoxide, on average 1.4-fold, in both epidermal and stelar cells in the elongation zone of *rbohD* compared with that in the WT and *gork1-1* after 1 h of hypoxia (Fig. 9). Comparing the superoxide concentration in the elongation zone in *rbohD* with that in the WT and *gork1-1*, epidermal and stelar cells had 1.3- and 1.8-fold higher superoxide concentrations than the same type of cells in the WT (Fig. 9). Seventy-two hours of hypoxic stress also induced the highest superoxide accumulation in *rbohD* compared with the WT and *gork1-1* in both elongation and mature zones (Fig. 9).

**Fig. 10.**
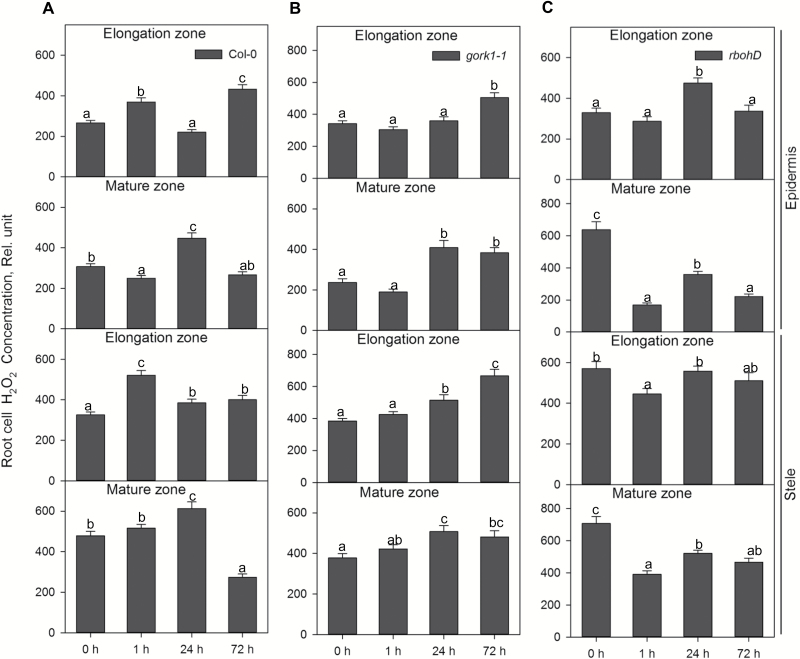
The time dependence of the relative H_2_O_2_ concentration in the elongation and mature zone in the root epidermis and stele of Col-0 (A), *gork1-1* (B), and *rbohD* (C) in response to hypoxia. Relative H_2_O_2_ concentration was calculated by the fluorescence integrated density using Image J software. Data are the mean ±SE [*n*=180–270; 20–30 cells analysed for at least nine individual roots (biological replicates)]. Different lower case letters indicate a significant difference at *P*<0.05.

The distribution of H_2_O_2_ in the *rbohD* mutant was found to be zone specific (Fig. 10; Supplementary Fig. S3). In the mature root zone of *rbohD*, the H_2_O_2_ concentrations in epidermal and stelar cells were first decreased dramatically after 1 h of hypoxia then increased significantly after 24 h, and finally declined remarkably after 72 h of hypoxia (Fig. 10C). In the mature zone of *gork1-1*, there were no changes after 1 h of hypoxia relative to control; however, a significant increase in H_2_O_2_ was found after 24 h and 72 h of hypoxia (Fig. 10B). In both the elongation zone and mature zone, the H_2_O_2_ concentration was increased more in the stele than in the epidermis in both mutants after 1, 24, and 72 h of hypoxia (Fig. 10B, C). In the WT, similar trends were also found after 1 h and 24 h of hypoxia, but not after 72 h (Fig. 10A).

## Discussion

### Preventing K^+^ leak by knocking out GORK channels results in a hypoxia-tolerant phenotype

To the best of our knowledge, no studies have been conducted focusing on the role of GORK channels in relation to hypoxia stress. Here we report that knocking out GORK channels results in a waterlogging-tolerant phenotype in *Arabidopsis,* with gork1-1 plants showing better shoot growth and being able to complete the life cycle (Fig. 2A). This quick switch from vegetative to reproductive growth suggests a more positive response in *gork1-1* to waterlogging stress than in *rbohD* and the WT which have significantly reduced shoot fresh weight and low chlorophyll content (Fig. 2B, D). The above observation is further corroborated by the fact that the GORK channel transcript levels in the WT were reduced by 4-fold when exposed to 24 h of hypoxia (Fig. 1A). The phenotypic observations were also consistent with higher amounts of cellular K^+^ in the epidermis and stele of *gork1-1* than in those of the WT after 24 h of hypoxia (Fig. 4A, B), as well as with the smaller hypoxia-induced K^+^ efflux from both the elongation and mature zone of *gork1-1* roots (Fig. 8). These findings are in good agreement with observations that the ability of roots to maintain cytosolic K^+^ homeostasis is a hallmark of plant acclimation to hypoxia in *Vitis riparia* (Mugnai *et al.*, 2011). Compared with *rbohD* and the WT, *gork1-1* lacks a major pathway for K^+^ efflux and thus presumably maintains more K^+^ in the cytosol when exposed to O_2_-deficient conditions. Taken together, these results suggest that preventing hypoxia-induced K^+^ leak is one of the key determinants of hypoxia tolerance in Arabidopsis.

Low O_2_ can limit the supply of ATP to the plasma membrane H^+^-ATPase, reducing membrane potential and impairing ion transport processes, thus altering nutrient status such as K^+^ homeostasis and further affecting cell metabolism (Bailey-Serres and Voesenek, 2008; Elzenga and van Veen, 2010; Shabala *et al.*, 2014). The low energy status under O_2_-deficient conditions leads to a substantial depolarization of the plasma membrane potential to −70 mV to −80 mV (Zeng *et al.*, 2014) which impairs K^+^ uptake through inward-rectifying channels and increases K^+^ efflux via depolarization-activated channels such as GORK channels in Arabidopsis (Véry *et al.*, 2014). A dramatic decline of ATP availability leads to cytosolic acidification (Felle, 2005) that is thought to active tonoplast-located V-ATPase for counteracting this under anoxic conditions (Dietz *et al.*, 2001; Koizumi *et al.*, 2011). Further studies found that vacuolar H^+^-pyrophosphatases (H^+^-PPases) can be activated when there is insufficient V-ATPase, arguing that this switch is beneficial to roots under anoxic treatment (Greenway and Gibbs, 2003). As more evidence on the signalling function of K^+^ in plant adaptive responses to stress start emerging (Anschutz *et al.*, 2014), the question of whether GORK channel-mediated K^+^ fluxes play a signaling role reprogramming plant metabolic pathways under hypoxia remains a matter of the future studies.

### Reduced NADPH oxidase activity results in a hypoxia-sensitive phenotype

When roots are under O_2_-deficient conditions, ROS can be produced, both in mitochondria and also in the apoplastic space by the plasma membrane NADPH oxidase. Cell wall-associated peroxidases (POXs) and oxalate oxidases (Kawano, 2003; Rhoads *et al.*, 2006) may be also involved. ROS not only react with a large variety of biomolecules causing irreversible damage (Moller, 2001; Dolferus *et al.*, 2003; Taylor *et al.*, 2004) but also alleviate stress-induced damage and participate as active players in the stress signalling cascades (Alvarez *et al.*, 1998; Dalton *et al.*, 1999; Miller *et al.*, 2010; Ray *et al.*, 2012; Shabala and Pottosin, 2014). ROS production under O_2_ deprivation conditions was found in many species, and increased ROS was accompanied by up-regulation of *RBOHA*, *RBOHB*, and *RBOHD* (Branco-Price *et al.*, 2005; Liu *et al.*, 2005; Marino *et al.*, 2011). This was proved in our work, where 1 h of hypoxia caused up to 3-fold increases in the superoxide level in epidermal and stelar cells in both the elongation and mature zones in WT, *gork1-1*, and *rbohD*, except for the stelar cells of the elongation zone in the WT (Fig. 9A, C). It was found that H_2_O_2_ serves as a signalling molecule in the ROP (RHO-like small G-protein of plants) signal transduction pathway in Arabidopsis via an NADPH oxidase mechanism (Baxter-Burrell *et al.*, 2002). In addition, Pucciariello *et al.* (2012) also found that ROS are required to regulate a set of heat shock proteins (HSPs) and ROS-related transcription factors (TFs) with the activation of NADPH oxidase. The distribution of H_2_O_2_ in the *rbohD* mutant in the root mature zone was significantly decreased relative to the WT after 1 h of hypoxia (Fig. 10A, C), which suggested that a lack of RBOHD affects the ROS pathway then subsequently leads to the sensitive to hypoxia phenotype.

A previous study has shown that the *rbohd* mutant is slightly smaller than the WT under normal growth conditions (Torres *et al.*, 2002) but provided no quantitative data to prove that the observed effect was significant. In our work, no significant difference was found between these two genotypes using Duncan’s multiple range test (Fig. 2E). This is consistent with Chen *et al.* (2015*a*) who also found no statistically significant difference in plant size between these two genotypes. Thus, it appears that the lack of *rbohD* activity under normal conditions may be compensated by either other NADPH oxidase isoforms or other sources of ROS production, whereas this role cannot be substituted under hypoxia stress conditions.

Recent studies identified RBOHs as key signalling nodes in the network of plants integrating multiple signal transduction pathways (Suzuki *et al.*, 2011). ROS are key molecules that signal the low K^+^ status in plants (Ashley *et al.*, 2006), and continuous depletion of the cytosolic K^+^ pool may activate caspase-like proteases leading to programmed cell death (Demidchik *et al.*, 2010). Putative lysigenous aerenchyma development in Arabidopsis hypocotyls involved H_2_O_2_ and ethylene signalling in response to hypoxia (Muhlenbock *et al.*, 2007), but this remains to be confirmed and is not known for roots of this species. In the present study, knocking out *RBOHD* in Arabidopsis showed significant growth penalties relative to the WT after 3 weeks waterlogging stress (Fig. 2). The K^+^ concentration in the root epidermis in the mature zone was higher than that in the WT after 24 h of hypoxia (Fig. 4A, C) while in the same cells the H_2_O_2_ accumulation was less than in the WT (Fig. 10A, C). Thus, we speculate that RBOHD activity may be essential to activate the ROS-sensitive pathway of K^+^ leak from Arabidopsis root cells under hypoxia stress.

It should also be kept in mind that NADPH oxidase is not the only source of ROS, and the mitochondrial electron transport chain also plays a crucial role in hypoxia-induced ROS accumulation. Also, other RBOH isoforms (10 in total; Sagi and Fluhr, 2006) might contribute to ROS accumulation. This can explain the rather complex time-dependent kinetics of H_2_O_2_ accumulation reported in this work (Fig. 10) and some possible inconsistencies with changes in transcript levels for *rbohD*.

### Knocking out RBOHD may interfere with stress-induced Ca^2+^ signalling

The production of ROS by RBOHs is identified to be synergistically activated by the binding of Ca^2+^ to EF-hand motifs as well as Ca^2+^-dependent phosphorylation (Kurusu *et al.*, 2015). NADPH oxidase-mediated production of ROS has also been suggested to play crucial roles in regulating adaptation to different stresses in several plant species. In maize, H_2_O_2_ was generated in mitochondria by anoxic stress and accompanied by elevation of [Ca^2+^]_cyt_ (Subbaiah *et al.*, 1998). Hypoxia increases the [Ca^2+^]_cyt_ that leads to activation of plasma membrane NADPH oxidase. H_2_O_2_ generated by NADPH oxidase can then activate ROS-sensitive depolarization-activated Ca^2+^-permeable cation channels (DACCs), leading to more apoplastic Ca^2+^ entering the cytosol (Lecourieux *et al.*, 2002; Shabala *et al.*, 2014). A clear trend of hypoxia-induced accumulation of Ca^2+^ was found in all root cell types of the three genotypes studied here (Fig. 5). Comparing Ca^2+^ concentration in the epidermis in the WT and *rbohD*, the increase in Ca^2+^ was much less pronounced in the latter (Fig. 5), which may indicate an important role for RBOHD as the source of Ca^2+^ signal. Novel studies on ROS waves under different abiotic stresses validated that RBOHD acts as an engine for propagation of a rapid systemic autopropagating wave of ROS production by the accumulation of H_2_O_2_ (Gilroy *et al.*, 2014). Likewise, a recent report demonstrated that Ca^2+^-dependent protein kinase 5 (CPK5) is key for the propagation of the ROS wave in plants (Dubiella *et al.*, 2013), which underlines that RBOH proteins may interfere with Ca^2+^ waves and ROS signalling. Based on the present result, we suggest that RBOHD may interfere with Ca^2+^ signalling under hypoxic stress.

## Conclusion

This study highlighted the essential roles of GORK channels and NADPH oxidase as components of plant adaptive response mechanisms to hypoxia in Arabidopsis roots. A loss-of-function mutant of the GORK channel exhibits higher K^+^ accumulation compared with the WT in different zones and tissues under hypoxic stress. By preventing hypoxia stress-induced K^+^ leak, the *gork1-1* mutant showed higher waterlogging tolerance compared with the WT. Reduced NADPH oxidase activity resulted in the hypoxia-sensitive phenotype in the *rbohD* mutant, with a less pronounced increase of Ca^2+^ concentration and higher superoxide accumulation in the elongation zone compared with the WT. We suggest that RBOHD is a crucial component interfering with stress-induced Ca^2+^ signalling.

## Supplementary data

Supplementary data are available at *JXB* online

**Figure S1** . Effect of hypoxic stress on Ca^2+^ distribution in Arabidopsis roots.

**Figure S2** . Effect of hypoxic stress on superoxide distribution in Arabidopsis roots.

**Figure S3** . Effect of hypoxic stress on H_2_O_2_ distribution in Arabidopsis roots.

## Supplementary Material

supplementary_figures_S1_S3
