# Supplementary material for: Revealing the roles of GORK channels and NADPH oxidase in acclimation to hypoxia in Arabidopsis
Source: J Exp Bot. 2016 Oct 19;68(12):3191–204. doi: 10.1093/jxb/erw378 (PMC5853854; doi:10.1093/jxb/erw378)
Supplement: supplementary_figures_S1_S3 [file erw378_suppl_supplementary_figures_s1_s3.pdf]

Supplementary data

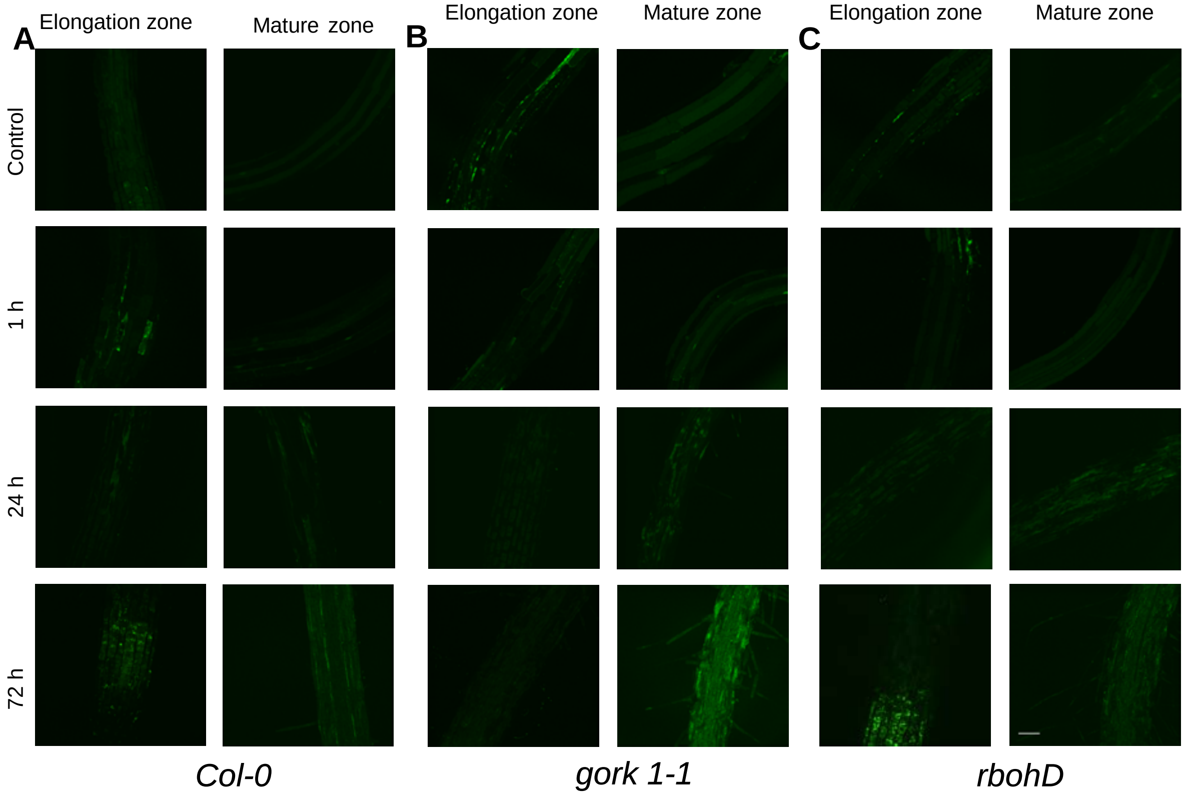

Supplementary Fig. 1. Effect of hypoxic stress on Ca^{2+} distribution in root elongation and mature zone in *Arabidopsis* wild-type (Col-0), *gork1-1* and *rbohD*. Representative images of root elongation and mature zone in Col-0 (A), *gork1-1* (B) and *rbohD* (C) under control and hypoxic treatment. Ten-day-old seedlings was stained with Calcium Green-5N and visualised with confocal imaging system. One out of nine typical images is shown for each line. Scale bar = 50 µm.

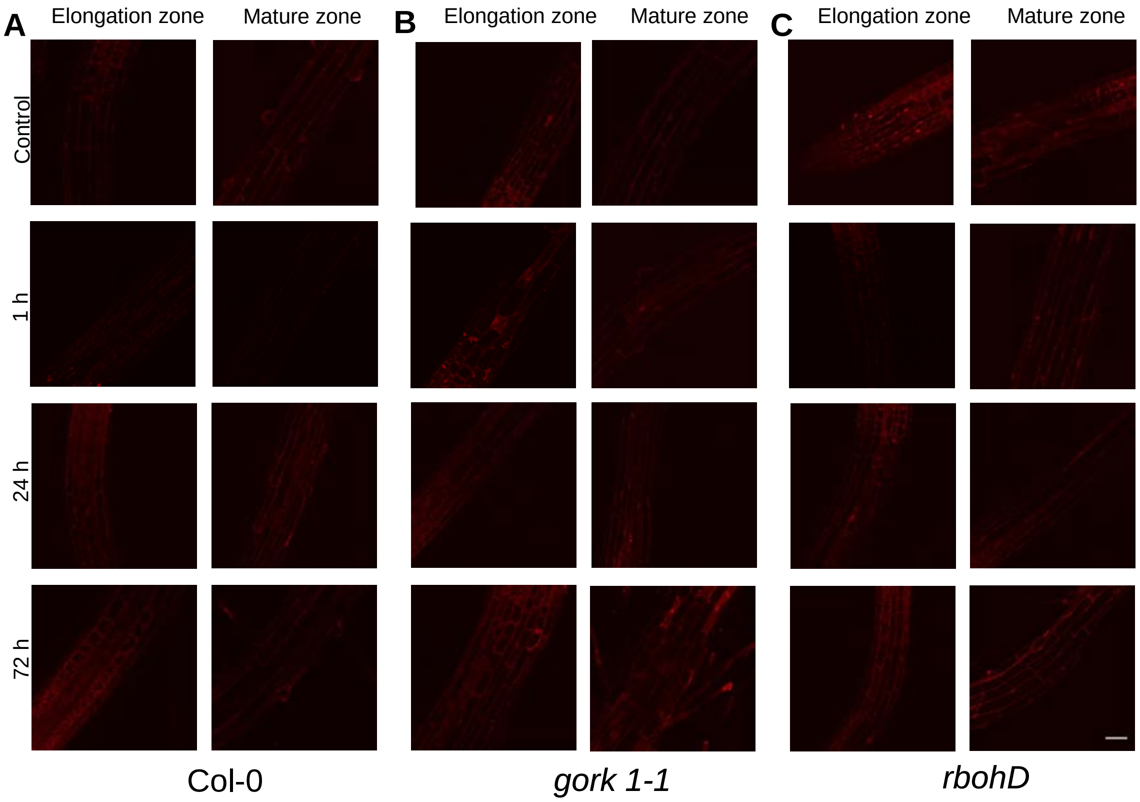

Supplementary Fig. 2. Effect of hypoxic stress on superoxide distribution in root elongation and mature zone in *Arabidopsis* wild-type (Col-0), *gork1-1* and *rbohD*. Representative images of root elongation and mature zone in Col-0 (A), *gork1-1* (B) and *rbohD* (C) under control and hypoxic treatment are shown. Ten-day-old seedlings was stained with superoxide indicator (DHE) and visualised with confocal imaging system. One out of nine typical images is shown for each line. Scale bar = 50 µm.

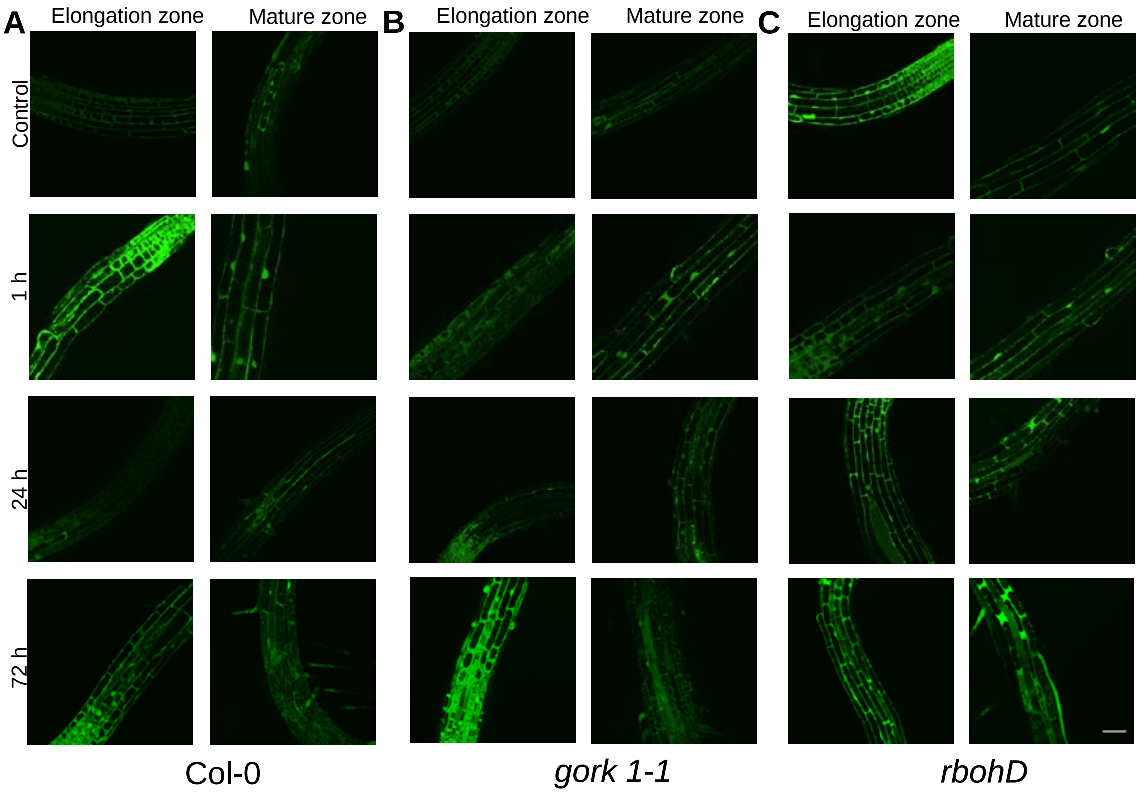

Supplementary Fig. 3. Effect of hypoxic stress on H_2O_2 distribution in root elongation and mature zone in *Arabidopsis* wild-type (Col-0), *gork1-1* and *rbohD*. Representative images of root elongation and mature zone in Col-0 (A), *gork1-1* (B) and *rbohD* (C) under control and hypoxic treatment. Ten-day-old seedlings was stained with H_2O_2 indicator DCF and visualised with confocal imaging system. One out of nine typical images is shown for each line. Scale bar = 50 µm.
